# Combining immune checkpoint inhibition plus tyrosine kinase inhibition as first and subsequent treatments for metastatic renal cell carcinoma

**DOI:** 10.1002/cam4.4679

**Published:** 2022-03-18

**Authors:** Yuanquan Yang, Sarah P. Psutka, Anish B. Parikh, Mingjia Li, Katharine Collier, Abdul Miah, Sherry V. Mori, Megan Hinkley, Scott S. Tykodi, Evan Hall, John A. Thompson, Ming Yin

**Affiliations:** ^1^ Genitourinary Oncology Section, Division of Medical Oncology The Ohio State University Comprehensive Cancer Center – James Cancer Hospital Columbus Ohio USA; ^2^ Department of Urology University of Washington School of Medicine Seattle Washington USA; ^3^ Department of Pharmacy The Ohio State University Wexner Medical Center Columbus Ohio USA; ^4^ Division of Medical Oncology Fred Hutchinson Cancer Research Center, University of Washington Seattle Washington USA

**Keywords:** immune checkpoint inhibitor, immunotherapy, renal cell carcinoma, retrospective review, tyrosine kinase inhibitor

## Abstract

**Background:**

Immune checkpoint inhibitor/tyrosine kinase inhibitor (ICI/TKI) combinations are a new standard of care for the initial treatment of metastatic renal cell carcinoma (mRCC). Their efficacy and toxicity beyond the first‐line setting remain poorly defined.

**Methods:**

We retrospectively reviewed charts for 85 adults with mRCC of any histology receiving combination of ICI/TKI in any line of treatment at two academic centers as of 05/01/2020. We collected clinical, pathological, and treatment‐related variables. Outcomes including objective response rate (ORR), progression‐free survival (PFS), and toxicity were analyzed via descriptive statistics and the Kaplan–Meier method.

**Results:**

Patients received pembrolizumab, nivolumab, avelumab, or nivolumab–ipilimumab, with concurrent use of sunitinib, axitinib, pazopanib, lenvatinib, or cabozantinib. Thirty‐three patients received first‐line ICI/TKI therapy, while 52 received ≥ second‐line ICI/TKI. The efficacy of ICI/TKI therapy decreased with increasing lines of treatment (ORR: 56.7%, 37.5%, 21.4%, and 21%; median PFS [mPFS]: 15.2, 14.2, 10.1, and 6.8 months, for first, second, third, and ≥ fourth line therapy, respectively). In the ≥ second‐line setting, ICI/TKI was most useful in patients who received ICI only, with an ORR of 50% and a mPFS of 9.1 months. Efficacy was limited in patients who received both TKI and ICI previously, with an ORR of 20% and a mPFS of 5.5 months. Overall, ≥ second‐line ICI/TKI was tolerable with 25 of 52 (52%) patients developing grade ≥3 adverse events.

**Conclusions:**

ICI/TKI combination therapy is feasible and safe beyond the first‐line setting. Prior treatment history appears to impact efficacy but has a lesser effect on safety/tolerability.

## Graphical abstract

ICI/TKI combination therapy is effective and safe beyond the first‐line setting. Prior treatment history appears to impact efficacy but has a lesser effect on safety/tolerability.

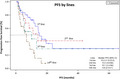

## 1 INTRODUCTION

In 2019, the US Food and Drug Administration (FDA) approved both pembrolizumab‐axitinib and avelumab‐axitinib for the first‐line (1L) treatment of metastatic renal cell carcinoma (mRCC). Each regimen consists of an immune checkpoint inhibitor (ICI) targeting either the programmed cell death protein 1 (PD‐1, pembrolizumab) or its ligand (PD‐L1, avelumab) in combination with axitinib, a tyrosine kinase inhibitor (TKI) targeting the vascular endothelial growth factor pathway. As such, these regimens represent rationally designed novel therapeutic combinations built upon earlier work showing individual efficacy of each class of drugs in RCC.^1^, ^2^, ^3^, ^4^, ^5^, ^6^, ^7^, ^8^ The FDA approvals were based on impressive clinical benefits seen in the Keynote‐426 and Javelin Renal 101 trials, with an unprecedentedly high objective response rate (ORR) of 52.5%–59.3% and median progression‐free survival (mPFS) around 13.8–15.1 months.^9^, ^10^, ^11^, ^12^ Since then, additional ICI/TKI combinations such as nivolumab–cabozantinib and pembrolizumab‐lenvatinib have been assessed in phase III clinical trials and consistently showed high efficacies in mRCC.^13^, ^14^ As a result, nivolumab–cabozantinib was approved by the FDA in 2020, while pembrolizumab‐lenvatinib gained FDA approval in 2021. An overview of the four ICI/TKI combinations seems to show comparable clinical outcomes, including ORR, PFS, and overall survival (OS) advantages over sunitinib, except avelumab‐axitinib, which is yet to demonstrate an OS benefit.

While ICI/TKI combinations have emerged as new 1L options for mRCC, it remains unclear how such combinations fit into the larger landscape of mRCC management, both in the 1L setting and beyond. Indeed, the most current iteration of the National Comprehensive Cancer Network Guidelines for mRCC lists 10 distinct 1L treatment options, and 14 recommended subsequent‐line options.^15^ This growing abundance of therapies has highlighted the dearth of prospective trial data comparing these various options and has ultimately compounded the lack of clarity regarding optimal treatment selection and sequencing in this disease. More importantly, it is plausible that ICI refractory patients may benefit from continuing ICI at the time of progression due to immunomodulatory effects of TKIs. The concept is supported by two early phase clinical trials showing impressive ORR of lenvatinib‐pembrolizumab and tivozanib–nivolumab in ICI‐pretreated patients.^16^, ^17^ The numerically higher ORR of ICI/TKI combinations (55.8%–62%) compared to histological data of second‐line (2L) TKI alone (18%–27%) suggest a synergy between ICI and TKI. Given the relative novelty of ICI/TKI combinations, very little is known about these regimens outside of recently reported clinical trials, and there remains a lack of knowledge to date regarding their use beyond the 1L setting (i.e., ≥2L). Herein, we aim to study the efficacy and toxicity of ICI/TKI combination therapy in real‐world clinical practice in an effort to further improve the outcomes of patients with mRCC.

## 2 METHODS

### 2.1 Patient population

We retrospectively collected data between 05/01/2015 and 05/01/2020 from The Ohio State University Comprehensive Cancer Center (OSU) and the University of Washington (UW)—Seattle Cancer Care Alliance. The study was approved by the Institutional Review Board and Ethics Committee at both institutions, and patient consent was waived. We collected data on 85 patients with mRCC of any histology who were at least 18 years of age and who had received at least two lines of ICI‐based systemic therapy, consisting of either pembrolizumab, nivolumab, avelumab, or nivolumab–ipilimumab, with concurrent TKI use including one of sunitinib, axitinib, pazopanib, lenvatinib, or cabozantinib. Although our study focused on ≥2L use of ICI/TKI, patients who received 1L ICI/TKI treatment were included as a comparison group. Patients were excluded if they received ICI/TKI therapy elsewhere.

### 2.2 Data and outcomes

Clinical data including patient demographics, histologic subtype, International Metastatic RCC Database Consortium (IMDC) risk group, RCC treatment history, ICI/TKI regimen, and line of therapy were recorded. Measurements of ORR, PFS, and safety/toxicity were used for the assessment of clinical outcomes. ORR was defined as best response by complete response (CR) and partial response (PR), while treatment response was evaluated by medical oncologists based on RECIST 1.1 criteria. Patients without measurable diseases were excluded from ORR analysis. PFS was defined as the time of ICI/TKI therapy initiation to the date of radiographic disease progression or death from any cause. For patients who had not yet progressed but were switched to another therapy (e.g., due to toxicity), PFS was censored at the date of the last evaluable tumor assessment prior to the treatment change. Safety and tolerability were determined by related descriptions in chart review and were assessed for grade ≥3 treatment‐related adverse events (AEs) based on the Common Terminology Criteria for Adverse Events Version 5.0.

### 2.3 Statistical analysis

Details regarding demographics, disease, treatment, and toxicity characteristics were summarized by descriptive statistics. PFS was estimated using a Kaplan–Meier survival curve. The Cochran‐Armitage trend test and Wilcoxon trend test were performed to assess the association of the lines of ICI/TKI treatment with ORR and PFS outcomes, respectively. All statistical procedures were performed using SAS 9.1 software (SAS, Inc.).

## 3 RESULTS

### 3.1 Patient characteristics

A total of 85 patients with mRCC were included, of which 65 were from OSU and 20 were from UW. Thirty‐three patients (39%) received 1L ICI/TKI and 52 patients (61%) received ≥2L ICI/TKI. The median follow‐up time was 11.3 months (range 1.7–66.0 months). Clinicopathological characteristics for the cohort are presented in Table 1. The median age was 60 years (interquartile range [IQR], 53.3–64.6) for the entire cohort, 60.5 years (IQR, 55.9–65.6) for patients with 1L ICI/TKI treatment, and 57.9 years (IQR, 51.1–63.9) for patients with ≥2L ICI/TKI treatment. The majority of the patients were male (81%, *N* = 69) and the most common histology was clear cell (79%, *N* = 67), while 12 (21%) patients had non‐clear cell RCC, including five papillary type, one chromophobe type, one translocation type, and five unclassified type.

**TABLE 1:** Patient characteristics

Parameters	Total (%)	1L (%)	≥2L (%)
Number	85	33	52
Median age (range)	60 (25.7–82.2)	60.5 (37.6–82.2)	57.9 (25.7–76.6)
Sex			
Male	69 (81.1)	26 (78.8)	43 (82.7)
Female	16 (18.9)	7 (21.2)	9 (17.3)
Histology			
Clear cell	67 (78.8)	27 (81.8)	40 (76.9)
Non‐clear cell	12 (14.1)	4 (12.1)	8 (15.4)
Papillary	5 (5.9)	0	5 (9.6)
Chromophobe	1 (1.2)	0	1 (1.9)
Translocation	1 (1.2)	0	1 (1.9)
Unclassified	5 (5.9)	4 (12.1)	1 (1.9)
Unknown	6 (7.1)	2 (6.1)	4 (7.7)
IMDC risk group			
Favorable	20 (23.5)	5 (15.2)	15 (28.8)
Intermediate	46 (54.1)	19 (57.5)	27 (51.9)
Poor	17 (20)	9 (27.3)	8 (15.4)
Unknown	2 (2.4)	0 (0)	2 (3.8)
Nephrectomy			
Yes	64 (75.3)	21 (63.6)	43 (82.7)
No	21 (24.7)	12 (36.4)	9 (17.3)
Metastatic site			
Lymph node	33 (38.8)	13 (39.4)	20 (38.5)
Lung	44 (51.8)	14 (42.4)	30 (57.7)
Liver	13 (15.3)	6 (18.2)	7 (13.5)
Bone	20 (23.5)	10 (30.3)	10 (19.2)
CNS	6 (7)	2 (6.1)	4 (7.7)
Muscle	4 (4.7)	1 (3)	3 (5.8)
Local tumor bed	3 (3.5)	1 (3)	2 (3.8)
Other kidney	11 (12.9)	8 (24.2)	3 (5.8)
Others	13 (15.3)	6 (18.2)	7 (13.5)

Abbreviations: 1L, first line; 2L, second line; IMDC, International Metastatic RCC Database Consortium; CNS, central nervous system.

In total, 20 patients (24%) were under favorable risk by IMDC criteria, 46 (54%) were intermediate risk, 17 (20%) were poor risk, and risk category was unknown for two patients (2%) due to missing data. The majority of the patients had undergone prior nephrectomy (75.3%, *N* = 64). The three most common metastatic sites were lung (51.8%), lymph node (38.8%), and bone (23.5%).

Table 2 shows the details of the seven different ICI/TKI combination treatments used in the patient population. In the 1L setting, 28 (84.8%) patients received either pembrolizumab/axitinib or avelumab/axitinib. In the ≥2L setting, 43 (82.6%) patients received either nivolumab–cabozantinib, nivolumab–axitinib, or pembrolizumab‐axitinib. The median number of prior lines of therapy was 2 (range, 1–10; IQR, 1–3). Prior to ICI/TKI combination therapy, 10 (19.2%) patients received dual‐ICI therapy with anti‐PD1 plus anti‐CTLA4 antibodies (i.e., nivolumab and ipilimumab), 27 (51.9%) patients received TKI and ICI sequentially, and 15 (29%) patients received TKI only.

**TABLE 2:** Treatment characteristics

Parameters	Total (%)	1L (%)	≥2L (%)
ICI/TKI line of therapy			
1	33 (38.8)	33 (100)	0 (0)
2	16 (18.8)	0 (0)	16 (18.8)
3	16 (18.8)	0 (0)	16 (18.8)
≥4	20 (23.6)	0 (0)	20 (23.6)
ICI/TKI regimen			
Pembrolizumab–axitinib	28 (32.9)	20 (60.6)	8 (15.4)
Nivolumab–cabozantinib	21 (24.7)	1 (3)	20 (38.5)
Nivolumab–axitinib	17 (20)	2 (6.2)	15 (28.8)
Nivolumab–ipilimumab–cabozantinib	7 (8.2)	1 (3)	6 (11.5)
Avelumab–axitinib	9 (10.6)	8 (24.2)	1 (1.9)
Nivolumab–sunitinib	2 (2.3)	1 (3)	1 (1.9)
Nivolumab–lenvatinib	1 (1.3)	0 (0)	1 (1.9)

Abbreviations: ICI, immune checkpoint inhibitor; TKI, tyrosine kinase inhibitor.

### 3.2 Efficacy

**FIGURE 1:**
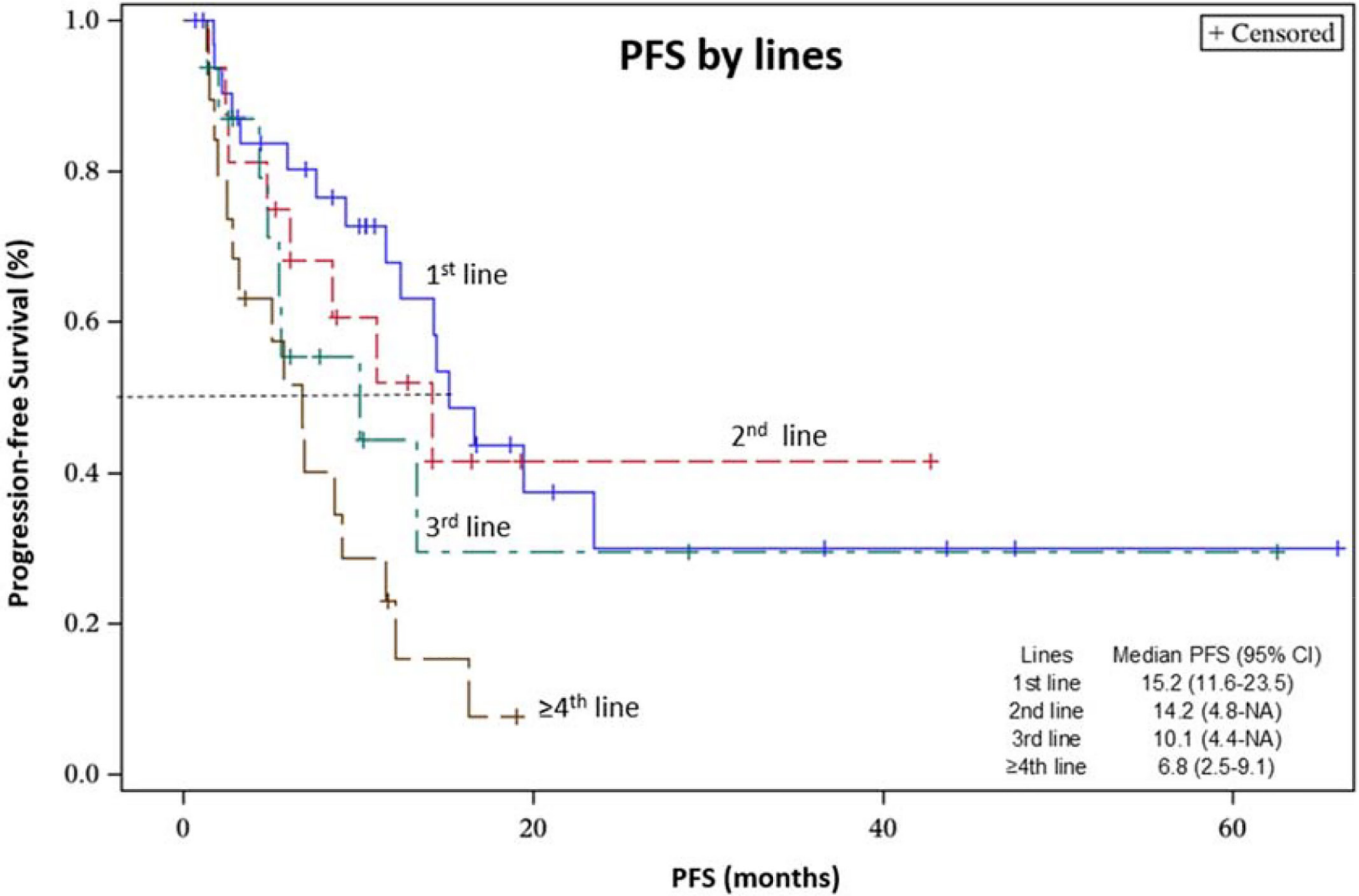
Progression‐free survival by lines of treatment

All 85 patients were eligible for PFS analysis with a mPFS of 11.6 months (95% CI, 8.5–14.5), while 79 (93%) patients had measurable diseases for ORR analysis, including 29 patients in the 1L setting and 50 patients in the ≥2L setting. A total of two CRs and 28 PRs were observed in the overall patient population. ICI/TKI had an ORR of 56.7% in the 1L setting, as compared to an ORR of 26.5% in the ≥2L setting. The efficacy of ICI/TKI therapy was negatively associated with the number of lines of treatment, for example the ORRs for 1L, 2L, third‐line, and ≥ fourth line therapies were 56.7%, 37.5%, 21.4%, and 21.0%, respectively (*p* trend <0.01). We also observed an analogous trend with decreasing PFS with increasing lines of therapy (*p* trend <0.01) with a mPFS of 15.2, 14.2, 10.1, and 6.8 months, respectively (Table 3; Figure 1).

**TABLE 3:** Objective response by line of therapy

Line of therapy	Patient no.	CR	PR	SD	PD	ORR (%)
First line	30	1	16	9	4	56.7
Second line	16	0	6	6	4	37.5
Third line	14	1	2	10	1	21.4
≥ Fourth line	19	0	4	9	6	21
Total	79	2	28	34	15	40

*Note*: Response not measurable in six patients, including three patients in first line.

Abbreviations: CR, complete response; ORR, objective response rate; PD, progressive disease; PR, partial response; SD, stable disease.

**FIGURE 2:**
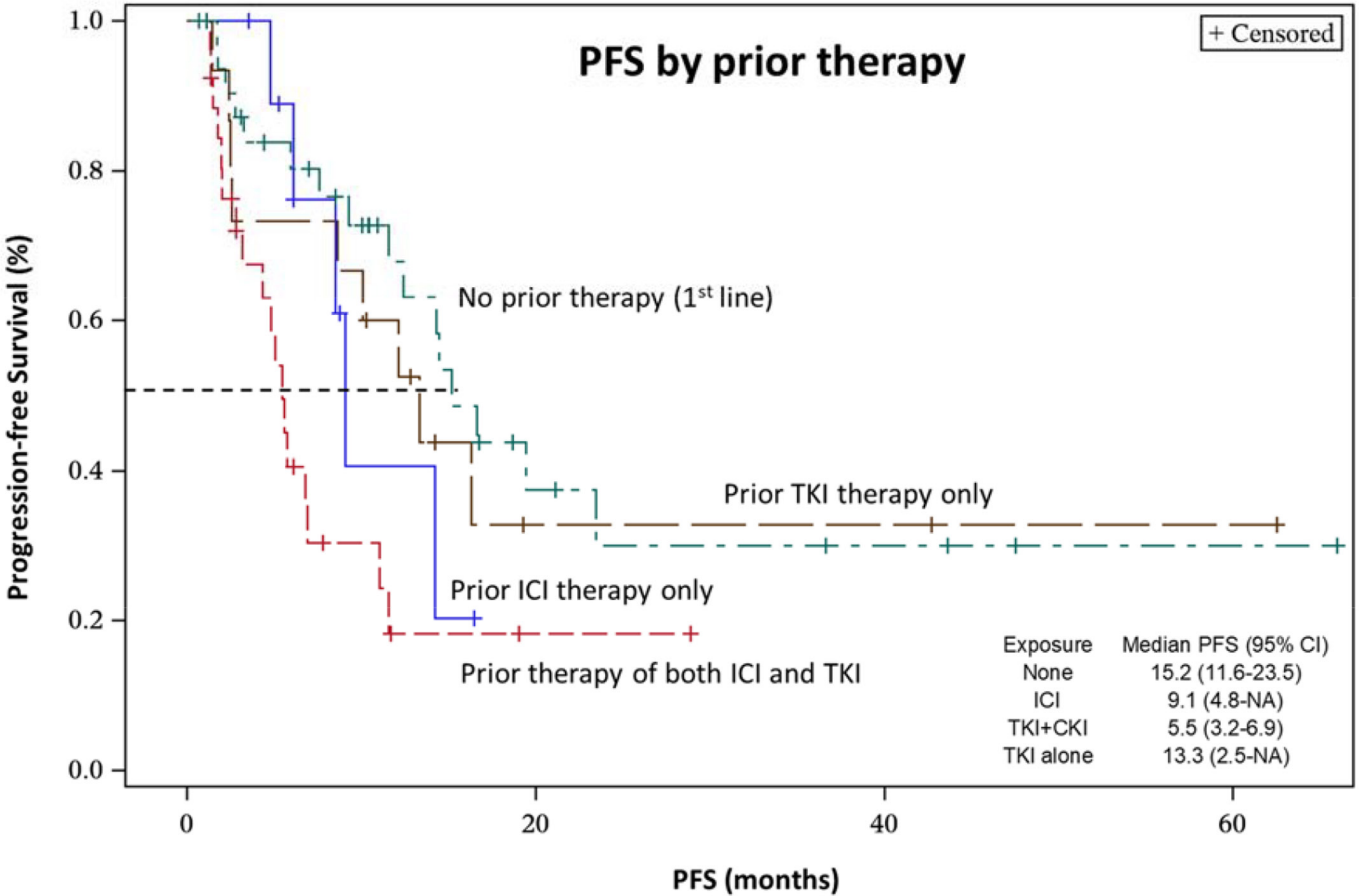
Progression‐free survival by prior treatment exposure

We attempted to assess the efficacy of ICI/TKI in non‐clear RCC. Three out of the four patients who received 1L and six out of the eight patients who received ≥2L ICI/TKI had evaluable responses. The ORRs were 0 and 33% (2 PRs), respectively. We then performed stratified analyses based on prior treatment exposure. In TKI‐naïve patients with prior ICI‐only therapy (nivolumab and ipilimumab), the ORR was 50% with a mPFS of 9.1 months (95% CI, 4.8—not reached). Conversely, in ICI‐naïve patients with prior TKI‐only exposure, the ORR was 20% with a mPFS of 13.3 months (95% CI, 2.5—not reached). In patients who had both prior TKI and ICI exposure followed by ≥2L TKI/ICI combination, the ORR was 20.8% with a mPFS of 5.5 months (95% CI, 3.2–6.9) (Table 4; Figure 2).

**TABLE 4:** Objective response in the ≥ second line by the types of prior therapy

Prior therapy	Patient no.	CR	PR	SD	PD	ORR (%)
Nivolumab–ipilimumab	10	0	5	4	1	50.0
Sequential TKI and ICI	24	0	5	13	6	20.8
TKI alone	15	1	2	8	4	20.0

*Note*: Response not measurable in three patients.

Abbreviations: CR, complete response; ICI, immune checkpoint inhibitor; ORR, objective response rate; PD, progressive disease; PR, partial response; SD, stable disease; TKI, tyrosine kinase inhibitor.

### 3.3 Safety of ≥2L ICI/TKI

Of the 52 patients who received ≥2L ICI/TKI, 27 (52%) had grade 3 or higher AEs. The most common ≥G3 AEs were anorexia (13.5%), diarrhea and hypertension (11.5% each), and fatigue (9.6%) (Table 5). Seventeen (32.7%) patients stopped ≥2L ICI/TKI for disease progression or death, while eight (16%) patients had to discontinue treatment due to ≥G3 AEs. AE rates of G3 or higher by the line of treatment were 62.5% (second line), 50% (third line), and 45% (≥ fourth line).

**TABLE 5:** Incidence of Grade ≥3 AE (%)

Total (*N* = 52)	27 (52)
Decreased appetite	7 (13.5)
Diarrhea/colitis	6 (11.5)
Hypertension	6 (11.5)
Fatigue	5 (9.6)
Nausea/vomiting	4 (7.7)
Musculoskeletal	2 (3.9)
Hematology toxicity	2 (3.9)
Liver toxicity	2 (3.9)
Endocrine	1 (1.9)
Hand–foot syndrome	1 (1.9)
Mucositis	1 (1.9)
Dyspnea	1 (1.9)
Skin toxicity	1 (1.9)
Other	3 (5.8)

## 4 DISCUSSION

This study is the largest case series to date examining off‐label use of ≥2L ICI/TKI in patients with mRCC. Our data suggest that ≥2L ICI/TKI is tolerable and feasible in salvage treatment of mRCC, and therefore warrants further investigations in larger clinical trials. The retrospective data from two major United States academic centers adds to the growing body of literature endorsing this approach.^18^, ^19^, ^20^

The treatment of mRCC is evolving from single‐agent therapy to dual‐agent therapy now. A recent meta‐analysis showed that immune‐based dual‐agent therapy tripled the CR rates and decreased the death risk by 26% in the first‐line setting, compared with TKI alone.^21^ The concept of ICI/TKI combination was first explored by combining PD‐1/PD‐L1 inhibitors plus a first‐generation TKI, such as pembrolizumab‐pazopanib, nivolumab–sunitinib, and nivolumab–pazopanib.^20^, ^22^ Although the efficacy was promising, 70%–90% patients experienced ≥G3 toxicities, leading to treatment discontinuation in approximately 20% of patients. Therefore, further development of these combinations was abandoned. Keynote‐426 (pembrolizumab‐axitinib), Javelin Renal 101 (avelumab‐axitinib), CheckMate‐9ER (nivolumab–cabozantinib), and CLEAR trial (pembrolizumab‐lenvatinib) were the pivotal trials that changed the standard practice of 1L therapy for mRCC, which adopted PD‐1/PD‐L1 inhibitors plus a second‐generation TKI and showed good efficacy with acceptable toxicities.^9^, ^10^, ^13^, ^14^ Those trial data showed ORR of 52.5%–71% and mPFS of 13.8–23.9 months in the 1L setting. Our real‐world upfront ICI/TKI experience demonstrates similar results in the 1L setting with an ORR of 56.7% and a mPFS of 15.2 months, comparable to those previously reported clinical trials.

Our study provides important evidence to guide ≥2L ICI/TKI therapy for metastatic RCC patients. Overall, we observed that ICI/TKI combination therapy demonstrated promising efficacy in the ≥2L setting. The ORR of our study was 37.5% for the second‐line and 27% for all subsequent lines combined, which is numerically similar or higher than the ORRs observed by other standard of care options in the 2L setting (43% for lenvatinib plus everolimus, 25% for nivolumab, 23% for axitinib, and 17% for cabozantinib).^4^, ^23^, ^24^, ^25^ The ORR is lower than that from a recently published retrospective series by Laccetti et al., which consisted of 48 mRCC patients who received ≥2L ICI/TKI in which an ORR of 51% was observed.^19^ This difference in ORR may be due to differences in 1L therapies utilized. For example, in the Laccetti et al. study, most patients received 1L TKI or ICI monotherapy before ≥2L TKI/ICI combination therapy, whereas almost half of the patients in our study had already received both TKI and ICI therapies sequentially. Unfortunately, further comparisons based on lines of therapy or treatment exposure are not possible because this information was not reported by Laccetti et al.

As expected, the current cohort demonstrated that the efficacy of ICI/TKI decreased with increasing lines of therapy, which could correlate with the accumulation of treatment‐related resistance by tumor genetic or epigenetic alterations. We observed a dismal PFS curve for ≥ fourth line group which is distinct from the other groups. This observation could be informative in clinical decision‐making, where clinical trials with novel agents should be strongly considered rather than continued FDA‐approved treatments.

Efficacy analyses based on prior treatment exposure are valuable to direct appropriate sequential treatment. For example, it is unclear whether patients will benefit more from upfront ICI/TKI followed by nivolumab–ipilimumab or vice versa. The ICI combination of nivolumab–ipilimumab is a standard 1L option in mRCC with a reported ORR of 42%, and it is also frequently used off‐label in the ≥2L setting. In our cohort, 2L ICI/TKI induced an impressive ORR of 50% with a durable mPFS of 9.1 months in the subgroup of patients (*n* = 10) who had previously only received 1L nivolumab–ipilimumab and were therefore TKI naive. These results are consistent with the results of a phase II study of ≥2L pembrolizumab‐lenvatinib in ICI‐pretreated patients, which demonstrated an ORR of 55.8% and a mPFS of 12.2 months.^17^ In contrast, ≥2L nivolumab plus ipilimumab demonstrated an ORR of approximately 20% in ICI‐pretreated patients with a mPFS of 4–5 months.^26^, ^27^, ^28^, ^29^, ^30^ Taken together, these results support the sequential use of 1L nivolumab–ipilimumab followed by 2L ICI/TKI therapy. The apparent synergy may be attributed to the immunomodulatory effects of TKI therapies which results in the resensitization of tumors to ICI.^31^, ^32^ Another subgroup that may derive benefit from ≥2L ICI/TKI are those ICI‐naïve patients who received prior TKI‐only therapy. Although the ORR was 20% in the ICI‐naïve group (*n* = 15), at last follow‐up, eight (53.3%) patients had stable disease, resulting in a durable PFS (median = 13.3 months). Our data suggest that the efficacy of ≥2L ICI/TKI was substantially reduced in patients who had previously been exposed to both TKI and ICI therapy with an ORR of only 20.8% and a mPFS of 5.5 months, similar to previously reported efficacy metrics observed with ≥2L TKI monotherapy.^24^ Taken together, our data suggest that the type of prior treatment might have an impact on the effectiveness of ≥2L ICI/TKI. The patients who received either TKI or immunotherapy but not both appear more likely to derive benefit from ≥2L ICI/TKI.

The efficacy of ICI/TKI in patients with non‐clear cell histology is unknown. There were 12 (21%) patients with non‐clear cell RCC in our cohort, and only nine patients were available for measurement of treatment response. Only two patients who received ≥2L ICI/TKI had PRs and both responders had unclassified histology. Although we did not observe objective response in the 1L setting, this sample size is too small to permit any substantive conclusions to be drawn and hence warrants further evaluation.

Finally, our study suggested that ≥2L ICI/TKI was safe and tolerable. Fifty‐two percent of the patients experienced ≥G3 AEs during treatment of ≥2L ICI/TKI. The ≥ grade 3 AE rate is lower than the rate of 71.2%–82.4%, which was reported in the phase III trials of 1L ICI/TKI.^9^, ^10^, ^13^, ^14^ No new safety signals were detected. Only 21% of patients stopped treatment due to toxicity or intolerance, whereas the majority of the patients (60%) discontinued treatment due to progressive disease.

### 4.1 Limitations

Our study has several limitations that must be acknowledged. First, it is limited by the inherent biases related to the retrospective study design. The objective response and toxicity assessments were not as rigorous as prospective studies. Nevertheless, the nearly identical response rate and mPFS to those receiving 1L ICI/TKI therapy suggest comparable clinical outcomes as observed in prospective studies. Second, the modest sample size did not allow us to further evaluate the data stratified by different ≥2L ICI/TKI combinations, to perform statistical comparisons among different subgroups, or to determine the impact of confounding factors. Although a trend test for efficacy by line of therapy was performed, our data are largely descriptive. Third, we did not present OS as a study endpoint because such analyses would be heavily biased due to heterogeneity of treatment and the timing of ICI/TKI initiation.

### 4.2 Conclusion

Overall, our data suggest that ≥2L ICI/TKI is safe and feasible in the treatment of mRCC that has progressed following initial systemic therapy. The hypothesis generated from our retrospective study was that the clinical benefit of salvage ICI/TKI was most pronounced in patients who had previously received either ICI or TKI treatment. In contrast, the clinical benefit of ≥2L ICI/TKI in patients who had previously received both ICI and TKI treatments was modest. Phase III trials to examine optimal treatment sequences such as PEDIGREE (NCT03793166), CONTACT‐03 (NCT04338269), and TiNivo‐2 (NCT04987203) are currently ongoing.

## CONFLICT OF INTEREST

The authors declare no conflict of interest.

## AUTHOR CONTRIBUTIONS

**Ming Yin**, **Sarah P. Psutka:** Conception and design. **Ming Yin**, **Sarah P. Psutka**, **Anish B. Parikh**, **Mingjia Li**, **Katharine Collier**, **Abdul Miah**, **Sherry V. Mori**, **Megan Hinkley**, **Yuanquan Yang**: Acquisition of data. **Ming Yin**, **Sarah P. Psutka**, **Yuanquan Yang:** Analysis and interpretation of data. **Ming Yin**, **Sarah P. Psutka**, **Yuanquan Yang**: Drafting of manuscript. All authors: Critical revision of manuscript. **Ming Yin:** Statistical analysis. **Ming Yin**: Supervision.

## ETHICS STATEMENT

Ethical approval was not sought from an institutional review board or ethics committee prior to commencing this study.

## Data Availability

The data that support the findings of this study are available on request from the corresponding author. The data are not publicly available due to privacy or ethical restrictions.
